# Glands

**Published:** 1888-09

**Authors:** C. W. Spalding

**Affiliations:** St. Louis


### GLANDS.

C. W. Spalding, M. D., St. Louis.

The functions of glands are among the most interesting and important of the organic processes of the animal body. They rank next to the vital organs in importance, and any interference with their functions is a serious matter, and with regard to some of them is followed by disastrous consequences.

The glands of the human body are very numerous, and may be divided into several kinds, the chief divisions being the true glands, the lymphatic or conglobate glands and the ductless glands.

Names are intended to be expressive of the quality of a thing or the function of an organ. If this rule had been followed in the naming of glands none but the true glands would have been entitled to the appellation. But they were all called glands before their respective functions were well understood, and having once found place in our literature under this name it was not advisable to change the name of either class, but to otherwise designate them according to their respective functions. Certainly those organs which elaborate and give forth all the amazing varieties of animal juices, which, notwithstanding their variety, are all drawn from the same source—the blood—should be called glands.

These are the true glands, and though the microscope shows scarcely a difference in the form and structure of the cells composing their tissues, yet the diversity of their secretions is something marvelous. There is no saliva in the blood, no gastric juices, no milk, no semen. The elements of these substances, even to the life spirit of the seminal germ, are in the blood, but the secretions themselves are elaborated in the glands by a cell-action peculiar to each variety.

Through the agency of the true glands, nature carries on one of her most wonderful operations. By this means the germ of the soul of man, as well as that of his body, is elaborated from the masculine blood. In the case of the lower animals that have no soul their inmost life is developed in a similar way. The blood of the mother furnishes the elements out of which the body is constructed, but the germ of both the internal and external comes only through the father.

The true glands may be again divided into secretory and excretory; the former supplying fluids useful for the purposes of the body, and the latter taking from the passing blood injurious substances and removing them from the system. Of the former class we may mention the salivary, the mucous or muciparous, the gastric, peptic, pancreatic, biliary: Peyer's, Brunner's, and Lieberkuhn's glands in the alimentary canal.

The principal ones found in the skin are the sebaceous, sudoriferous, cenemenous, and the meibomian. In other parts of the body are the prostate, the mammary, the synovial or mecelagmous, the lachrymal, and in the testes the seminal, through whose agency the species are propagated, while the brain itself may be considered as being of the nature of a gland, deriving from the blood as it does the nervous force for the vital purposes of the body.

The lymphatic glands, also called conglobate, are classed as absorbent, but they also have functions that are not clearly understood.

The ductless glands are the thyroid, the thymus, the supra-renal capsules, and the spleen.

Glands vary in size from about four pounds in weight to a size so small as to be visible to natural vision only when fully distended.

The largest gland of the human body is the liver. This immense gland has secreting functions that are peculiar. It secretes bile that is carried away by the bile ducts, and glycogen, which does not enter the bile ducts, but passes in some modified form directly into the blood stream through the hepatic veins. Besides its glandular functions the liver acts as the great storehouse of the carbo-hydrates, receiving and retaining these products of intestinal digestion, and giving them out into the blood in such proportion as the wants of the body require.

The dentist is more directly interested in the functions of the glands of the mouth. These in man are divided into salivary and mucous, and together these two classes furnish the mixed saliva of the mouth. The salivary consist of three pairs, the parotid, the submaxillary, and the sublingual. The mucous glands of the mouth are all of the same general character, and derive their names from their respective locations. The same is the case with the mucous glands of other parts, as tracheal, esophageal, uterine, vaginal, etc. The mucous glands of the mouth are the labial, lingual, buccal, palatine, and molar. On the tongue besides the mucous glands there are serous glands located near the circumvallate papilla which secrete saliva, and near the tip of the tongue are mixed (muco-salivary) glands which furnish a mixture of mucus and saliva.

The mucous glands are situated in the submucous tissue, and in form they are branched (recemore) tubular glands. Their secretions contain mucine and are acid in chemical reaction.

Pure saliva, which can only be obtained from the parotids, is always alkaline in reaction, and the mixed saliva of the mouth becomes acid only when the proportion of mucus is in excess, or when decomposition of epitheleum, salivary corpuscles or the remains of food takes place. The salivary corpuscles are a little larger than white blood corpuscles and are capable of molecular movements.

Other elements of saliva are calcium carbonate, from which tartar is chiefly formed; small quantities of sulphur, cyanide of potassium or soda and some organic substances of which that peculiar ferment, ptyaline, is the most important. Mucine is absent in pure saliva, and for this reason it is thin and clear.

The submaxillary always contains mucine, for the reason that in this gland we have both serous and mucous acini, the one secreting saliva and the other mucous, the serous, however, are far the most numerous.

The sublingual saliva, although containing much mucine, is strongly alkaline in reaction and abounds in salivary corpuscles. So the saliva of the mouth is a mixed substance derived from the secretions of salivary, mucous and other glands, and is more active than is that from any one gland. It should be an alkaline, or at least a neutral reaction. Its inorganic constituents besides those before mentioned are sodium and potassium chlorides, potassium-sulphate, alkaline and earthy phosphates and ferric-phosphate.

In diabetes mellitus it contains lactic acid, resulting from the decomposition of grape sugar. This acid dissolves the teeth causing diabetic dental decay. In new-born children there is a very scanty secretion of saliva until after two months of age. During this early period the diastasic ferment is not secreted by the submaxillary gland, nor by the pancreas; hence young children should not be fed on starchy food. For the digestion of milk the diastasic ferment is not required. As you well know, at a later period a copious secretion of saliva accompanies the eruption of the teeth.

At the red margin of the lips are a number of sebaceous glands which serve to lubricate the red surface of the lips.

Stimulation of the oral glands may be effected in various ways. If the facial nerve is stimulated at its origin, or the chorda-tympani at the peripheral end, a profuse flow of saliva follows.

On stimulation of the sympathetic a small flow of thick saliva occurs abounding in mucous and salivary corpuscles. These facts show that the nerves exercise an effect upon the secretions of all the glands of the mouth.

The secretion of saliva is largely a reflex act. When sapid substances are introduced into the mouth a copious flow of saliva takes place. The movements of mastication cause a similar flow, yet during the act of drinking, when this act is continued for any considerable time, the secretion of saliva ceases. The smell or the very thought of savory food causes a rapid flow of thin saliva, and thus the common expression, "the mouth waters," is literally true. During sleep there is no stimulation of nerves and consequently no secretion of saliva. If the mouth is held open during wakefulness the flow of saliva is as great if not greater than when the mouth is closed, but during sleep if the mouth is open it becomes dry. Again, if the nerves are divided, secretion stops, and in cases of paralysis the quantity secreted is greatly diminished.

The ductless glands elaborate substances which they secrete or excrete by infiltration.

The circulation of most of the glands of the body is very energetic, as the abundance of their blood-vessels and their frequent morbid alterations show.

